# The Global Anticoagulant Registry in the FIELD-Atrial Fibrillation (GARFIELD-AF): Exploring the changes in anticoagulant practice in patients with non-valvular atrial fibrillation in the Netherlands

**DOI:** 10.1007/s12471-016-0874-y

**Published:** 2016-08-25

**Authors:** V. ten Cate, H. ten Cate, F. W. A. Verheugt

**Affiliations:** 1 Laboratory for Clinical Thrombosis and Haemostasis, Cardiovascular Research Institute Maastricht, Maastricht, The Netherlands; 2 Department of Internal Medicine, Cardiovascular Research Institute Maastricht, Maastricht, The Netherlands; 3 P.C. Hooftstraat 188, 1071 CH Amsterdam, The Netherlands

**Keywords:** Atrial fibrillation, Anticoagulants, Stroke, Netherlands, Registries

## Abstract

There are over 385,000 cases of atrial fibrillation (AF) in the Netherlands, with over 45,000 new cases each year. Among other things, AF patients are at high risk of stroke. Patients are often prescribed oral anticoagulation, such as vitamin K antagonists (VKA), to mitigate these risks. A recently introduced class of oral anticoagulants, non-vitamin K antagonists (NOAC), is quickly gaining currency in global clinical practice. This study provides insight into the changes these new drugs will bring about in Dutch clinical practice.

GARFIELD-AF is a large-scale observational AF patient registry initiated in 2009 to track the evolution of global anticoagulation practice, and to study the impact of NOAC therapy in AF in particular. The registry includes a wide array of baseline characteristics and has a particular focus on: (1) bleeding and thromboembolic events; (2) international normalised ratio fluctuations; and (3) therapy compliance and persistence patterns. The results in this paper provide the baseline characteristics of the first cohorts of Dutch participants in this registry and discuss some of the consequences of the changes in anticoagulation practice.

Although VKA therapy remains overwhelmingly favoured by Dutch practitioners, NOACs are clearly gaining in popularity. Between 2011 and 2014, NOACs constituted an increasingly large proportion of prescriptions for oral anticoagulants.

The insights provided by the GARFIELD-AF registry can be used by healthcare systems to inform better budgetary strategies, by practitioners to better tailor treatment pathways to patients, and finally to promote awareness of the various available treatment options and their associated risks and benefits for patients.

## Introduction

Atrial fibrillation (AF) is the most commonly diagnosed cardiac arrhythmia, with over 385,000 estimated cases [1] in the Netherlands (5.5 % of the >55 year old age group). With approximately 45,000 new cases [1] each year, AF continues to grow at an alarming rate. Patients with AF have a fivefold risk of suffering from ischaemic stroke [2, 3], and are at considerably elevated risk for other thromboembolic events [4, 5]. Moreover, strokes are twice as likely to be lethal in patients with AF than in those without it [6]. The need for good thrombotic prophylaxis is evident.

The most widely used method of reducing stroke and other thromboembolic events in patients with AF is oral anticoagulation. Oral anticoagulants are predominantly subdivided into vitamin K antagonists (VKAs, e. g. warfarin) and non-vitamin K antagonist oral anticoagulants (NOACs), the latter subset comprising direct thrombin (dabigatran) and factor Xa inhibitors (rivaroxaban, apixaban, edoxaban). In the Netherlands, NOACs were only relatively recently approved: dabigatran in 2008, rivaroxaban in 2011, apixaban in 2011 and edoxaban in 2015. Although clinical trials indicate that NOACs are non-inferior, and in some respects superior (e. g. fewer intracranial haemorrhages), to VKAs [7], the number of patients treated with VKAs in the Netherlands still far exceeds the number of patients treated with NOACs.

In the Netherlands, a system of anticoagulant clinics has been in existence since the 1950s. This network of clinics spans the country and monitors the vast majority of patients on VKAs. Monitoring international normalised ratio (INR), issuing recommendations on dose adjustment and registration of side effects and bleeding complications have been routine practice for decades. When NOACs were introduced, the Health Council of the Netherlands recommended that these new agents should be carefully monitored, and that their efficacy and complications should be registered [8]. In spite of these recommendations, registration has not yet been formalised in the form of study protocols, and at present it remains uncertain whether proper monitoring of the complications of NOACs will be institutionalised in the same way it was for VKAs. This fact, combined with the slow uptake of NOACs in the Netherlands so far, has precluded Netherlands-based observational research into their relative risks and efficacy.

There are a number of reasons why it is important to complement experimental data with real-life or observational, data. Firstly, study subjects and protocols in experimental (e. g. randomised controlled trial) settings have often been found to inaccurately reflect the real patient population, i. e., key patient groups and typical care patterns are underrepresented [9]. Secondly, observational studies are able to incorporate a much wider range of potential risk factors, allowing for analysis of more complex comorbidity structures. Thirdly, and more pertinently for the prevention of thrombosis in AF in particular, like many other countries, the Netherlands could well be on the verge of a paradigm shift from VKAs to NOACs as the predominant type of thrombosis prophylaxis. Consequently, comparing adverse event outcomes between these two types of anticoagulation in a real-world setting will become increasingly more important for the provision of good healthcare for patients with AF.

## Methods

The GARFIELD-AF registry was initiated to overcome the dearth of information surrounding anticoagulation therapy, by collecting and aggregating global data on treatment patterns and clinical outcomes of non-valvular AF patients with ≥1 additional risk factors for stroke into one observational dataset [10]. The particular utility of this registry comes from its comprehensiveness, as: (1) the included baseline characteristics cover a wide array of potential epidemiological risk factors; (2) the dataset comprises both prospective and retrospective longitudinal cohorts; and (3) as of June 2014, it included patient data (target *n* = 55,000, current *n* = 41,677) from 35 different countries.

The data collected in the GARFIELD-AF registry will be particularly salient to those researchers and practitioners interested in: (1) the frequency of haemorrhagic and thromboembolic events in patients with AF, (2) INR fluctuations for patients treated with VKAs, (3) therapy compliance and persistence patterns [10]. Results relating to the above variables will be discussed in future GARFIELD-AF papers and are not covered by this paper.

### Patient inclusion criteria

All patients aged ≥18 years with a recent diagnosis (≤6 weeks) of non-valvular AF who have at least one additional risk factor for stroke and who have provided written informed consent are eligible for inclusion in the GARFIELD-AF registry. Patients to be included in the retrospective cohort (see cohort enrolment) should have been diagnosed within 6–24 months prior to enrolment into the registry.

The follow-up duration *D* for all patients is 2 years ≤ *D* ≤ 8 years. Patients for whom further follow-up is not expected or certifiably impossible are excluded from the registry, as are patients whose transient AF is secondary to a reversible cause.

### Cohort enrolment

There is a total of six cohorts, the first of which is retrospective, and the rest of which are prospective and sequential. All cohorts adhere to the same patient inclusion criteria, and are methodologically different only in terms of the period they cover. Patients included in the prospective cohorts (*n* = 50,000) are enrolled <6 weeks after diagnosis of AF, in five sequential cohorts. Retrospective patients (*n* = 5000) are enrolled 6–24 months after diagnosis. The results in this paper were derived from the retrospective cohort and the first three prospective cohorts, which span the periods December 2009 to October 2011, October 2011 to June 2013 and June 2013 to June 2014, respectively.

### Data entry

All patient data are entered into a database by practitioners. In the Netherlands, these are general practitioners and clinicians. Practitioners use the GARFIELD-AF electronic case report form (eCRF) for data entry. The eCRF is an online, multi-page questionnaire that addresses a host of patient characteristics. Practitioners are instructed to enter as much information about their patients as possible.

### Non-compliance measures

The GARFIELD-AF eCRF incorporates a number of entry options specifically relating to compliance and potential non-compliance indicators, e. g. dementia, low patient motivation or poor access to INR monitoring (for VKAs), as well as patient-indicated reasons for non-compliance, e. g. a perception of low stroke risk versus high bleeding risk, or certain dietary restrictions. This paper does not cover non-compliance related results.

## Results

As of June 2014, the Dutch sample included in GARFIELD-AF comprised 929 patients in three cohorts. Table 1 depicts a selection of descriptive statistics for the Dutch GARFIELD-AF cohorts 1 through 3. Table 2 depicts a number of baseline factors that may contribute to the occurrence of adverse events in patients with AF, such as smoking status and CHA_2_DS_2_-VASc and HAS-BLED scores.

**Table 1:** Baseline characteristics of GARFIELD-AF Dutch cohorts 1–3

Variable	Statistics	Cohort 1 retrospective patients (*n* = 93)	Cohort 1 prospective patients (*n* = 106)	Cohort 2 (*n* = 412)	Cohort 3 (*n* = 318)	Total without retrospective patients (*n* = 836)
Sex	*n* (% male)	93 (66.7)	106 (67.9)	412 (55.1)	318 (58.2)	836 (57.9)
Age at diagnosis	Mean (SD)	69.0 (9.3)	72.2 (8.7)	70.6 (10.2)	70.4 (9.9)	70.7 (9.9)
Type of AF diagnosed, *n* (%)	Permanent	5 (5.4)	5 (4.7)	8 (1.9)	6 (1.9)	19 (2.3)
	Persistent	10 (10.8)	7 (6.6)	32 (7.8)	7 (2.2)	46 (5.5)
	Paroxysmal	22 (23.7)	15 (14.2)	82 (19.9)	36 (11.3)	133 (15.9)
	New-onset	56 (60.2)	79 (74.5)	290 (70.4)	269 (84.6)	638 (76.3)
Baseline antithrombotic treatment, *n* (%)	VKA	66 (71.0)	74 (74.7)	285 (69.2)	218 (68.8)	577 (69.7)
	VKA+AP	8 (8.6)	14 (14.1)	54 (13.1)	29 (9.1)	97 (11.7)
	FXa	–	–	3 (0.7)	24 (7.6)	27 (3.3)
	FXa+AP	–	–	–	2 (0.6)	2 (0.2)
	DTI	1 (1.1)	–	6 (1.5)	16 (5.0)	22 (2.7)
	DTI+AP	–	–	2 (0.5)	4 (1.3)	6 (0.7)
	AP	11 (11.8)	6 (6.1)	32 (7.8)	10 (3.2)	48 (5.8)
	None	7 (7.5)	5 (5.1)	30 (7.3)	14 (4.4)	49 (5.9)
	Unknown	–	7	–	1	8

Data from the first three GARFIELD-AF prospective cohorts – cohort 1: Dec 2009–Oct 2011; cohort 2: Oct 2011–Jun 2013; cohort 3: Jun 2013–Jun 2014

*AP* aspirin, *FXa* factor Xa inhibitor, *DTI* direct thrombin inhibitor

**Table 2:** Risk factors of GARFIELD-AF Dutch cohorts 1–3

Variable	Statistics	Cohort 1 retrospective patients (*n* = 93)	Cohort 1 prospective patients (*n* = 106)	Cohort 2 (*n* = 412)	Cohort 3 (*n* = 318)	Total without retrospective patients (*n* = 836)
Diabetes, *n* (%)	Yes	20 (21.5)	20 (18.9)	81 (19.7)	58 (18.2)	159 (19.0)
Smoking status, *n* (%)	No	26 (41.3)	31 (37.8)	134 (45.0)	127 (51.8)	292 (46.7)
	Ex-smoker	26 (41.3)	37 (45.1)	119 (39.9)	75 (30.6)	231 (37.0)
	Current smoker	11 (17.5)	14 (17.1)	45 (15.1)	43 (17.6)	102 (16.3)
	Unknown	30	24	114	73	211
CHA_2_DS_2_-VASc score	*n* (missing)	87 (6)	103 (3)	388 (24)	302 (16)	793 (43)
	Mean (SD)	3.0 (1.3)	3.1 (1.5)	3.1 (1.5)	3.0 (1.5)	3.0 (1.5)
HAS-BLED score	*n* (missing)	48 (45)	59 (47)	194 (218)	161 (157)	414 (422)
	Mean (SD)	1.2 (0.9)	1.4 (1.0)	1.3 (0.9)	1.3 (0.9)	1.3 (0.9)

Data from the first three GARFIELD-AF prospective cohorts – cohort 1: Dec 2009–Oct 2011; cohort 2: Oct 2011–Jun 2013; cohort 3: Jun 2013–Jun 2014

**Fig. 1:**
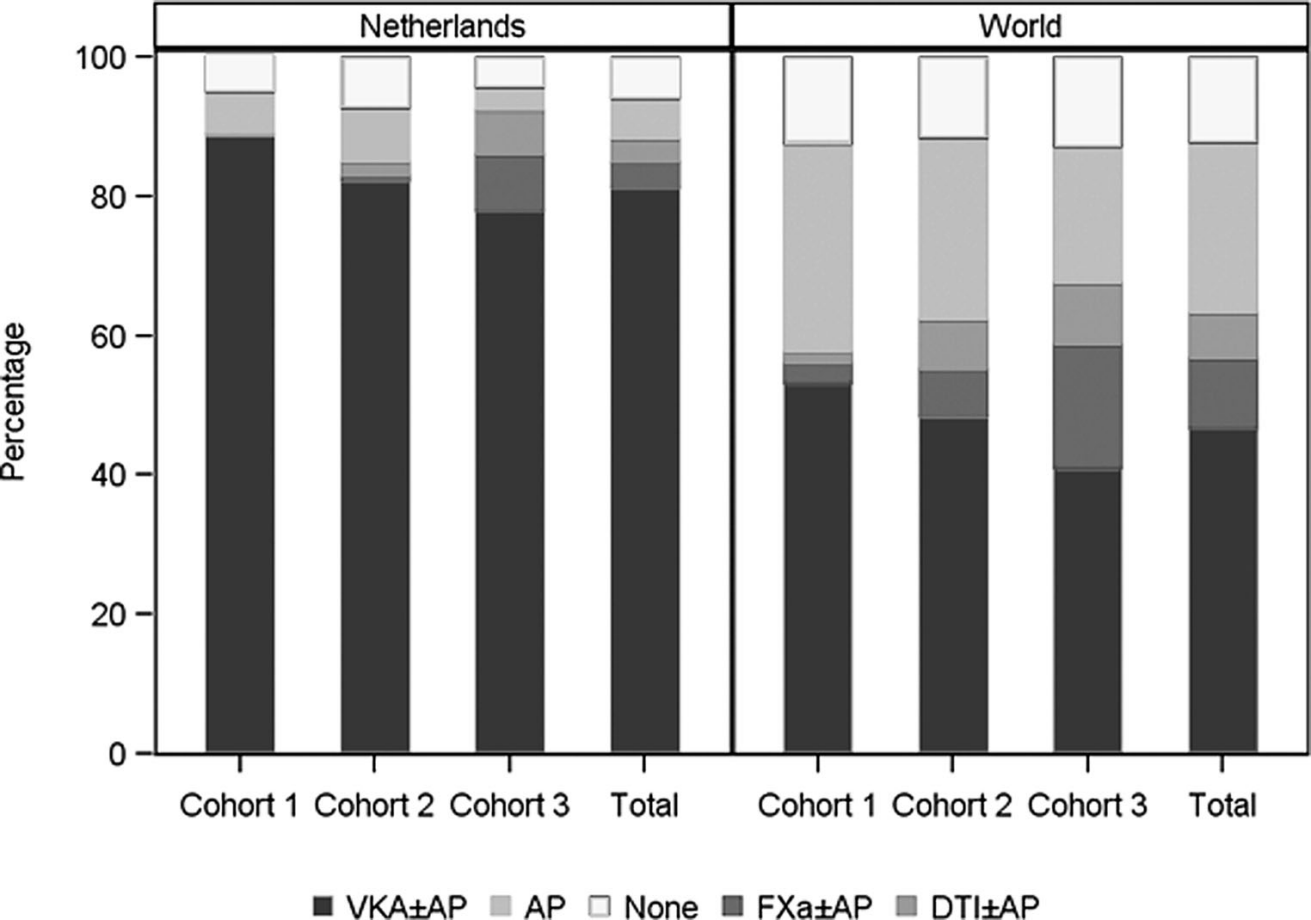
Treatment at diagnosis, by cohort

The data show that the patients entering the sequential cohorts are fairly consistent in age (Table 1) and CHA_2_DS_2_-VASc score (Table 2), with an average age of 71 years and risk score of 3 (SD 1.5). The majority of prospective patients were diagnosed with new-onset AF (73.6 %) at baseline, followed by paroxysmal AF (15.9 %). A large majority of prospective patients (81.4 %) were prescribed VKA, or VKA combined with aspirin, at baseline (Table 1). However, this proportion gradually diminished over time: from 88.8 % in the period 2009–2011 to 77.9 % in the period 2013–2014. This decrease occurred in unison with the gradual uptake of NOACs (Fig. 1), which went from 0 % in 2009–2011 to 14.5 % (NOACs or a combination of NOAC and aspirin at baseline) in 2013–2014 (Table 1). At the same time, the proportion of patients not receiving any form of antithrombotic medication is hardly affected, varying between 4.4 and 7.3 % in this country (Fig. 1). Worldwide, this group of subjects without antithrombotic medication averages around 12 % and that proportion, too, hardly changes in time (Fig. 1).

## Discussion

The introduction of new medications to the anticoagulation landscape has brought about changes in treatment patterns, which may result in confusion with regard to effective anticoagulation management among patients and practitioners without proper access to information. Now that the NOACs have been shown to be effective and safe for use in clinical trials, Phase IV research is needed to investigate the real-world impact of these new drugs. The availability of a large, variable-rich and non-interventional dataset such as GARFIELD-AF may be used to advance our understanding of how the various types of anticoagulation compare with one another in their uptake and in daily management by patients, and which are consequently most suitable for real-life scenarios.

The preliminary data, with a focus on the Netherlands in this manuscript, show remarkable changes over time, with substantial variation across countries. Within the Netherlands, a very gradual uptake of NOACs has been observed compared with many other countries, including its neighbour Belgium where only approximately 20 % of patients with AF are still on VKA therapy (data not shown). This rather striking contrast between the two countries may be explained by the presence of a network of anticoagulation clinics spanning the Netherlands; this situation is quite different in Belgium as well as many other countries around the globe.

Among the consecutive cohorts, it should be noted that a fairly fixed proportion of patients remain untreated; the proportion of patients on aspirin appears to diminish in time, which may in part be caused by a relatively larger proportion of patients who receive an NOAC. Due to the nature of this study we cannot, however, provide explanations as to the decision-making processes that underlie these apparent changes in prescriptions.

When compared with the pooled world data, it appears that many patients in the Netherlands are prescribed oral anticoagulants; usually they are prescribed VKA, in view of the data presented in this paper. Obviously, VKA therapy is challenging: it requires patient-specific titration and periodic readjustment of the prescribed dose throughout therapy to properly manage the patient’s prothrombin time, reflected by the INR. VKA therapy is reasonably effective and safe within an INR range of 2–3 (in the Netherlands, a slightly higher range of 2.5–3.5 was preferred until recently) [11], considering a time within therapeutic range (TTR) of >70 % as optimal. According to the Rosendaal linear interpolation technique, the average TTR (INR range 2.5–3.5) for long-term users in the Netherlands is 81 % [12]. The fact that VKA management requires frequent hospital visits and blood sampling may also impact in different ways on the inclination or ability of patients to initiate or continue therapy [13]. Finally, VKAs have a host of food-drug and drug-drug interactions [14], complicating their management still further. In light of these issues, NOAC therapy offers many practical advantages: NOACs are prescribed in fixed doses, do not require continuous monitoring and have fewer interactions with food and drugs. In spite of the potential advantages of NOACs there are still some hurdles, including the persistent risk of major bleeding complications while on oral anticoagulation [15]. NOACs remain under particular scrutiny in this regard [16, 17], owing to the absence of effective antidotes to factor Xa and direct thrombin inhibitors until recently [18]. However, this situation is changing; a dabigatran antidote, idarucizumab, was approved by the FDA at the end of last year and is currently registered in the Netherlands too [19].

Perhaps the biggest impediment to treatment efficacy is poor therapy compliance. Although non-compliance patterns are diverse (e. g. regular short gaps in medication-taking versus infrequent longer spells), the impact on treatment efficacy often amounts to the same negative result. This is particularly so for the NOACs, which have much shorter half-lives than VKAs [2]. Research indicates that suboptimal dabigatran adherence is associated with increased risk for all-cause mortality and stroke [20]. This underlines the pressing need to provide good information to patients, but also to organise integrated antithrombotic care for long-term users of anticoagulants. Integrated antithrombotic care has recently been recommended as standard care in the nationally endorsed National Standard of Integrated Antithrombotic Care 2.0 (LSKA 2.0) [21].

The GARFIELD-AF registry has spawned several research articles that attest to the usefulness of the dataset in informing better anticoagulation practices. An analysis of the characteristics of the first cohort (*n* = 10,614, spanning 19 countries, enrolment period December 2009 to October 2011) indicates that at the end of the VKA-only era, anticoagulant therapy was underused in patients at high risk of stroke and overused in those at low risk [22]. Another, more recent analysis confirmed these findings and also showed that more women than men were at moderate-to-high risk of stroke [23]. GARFIELD-AF is among the largest and longest-running of several recent large-scale observational registries charting anticoagulation use and outcomes in AF.

National initiatives, such as the PINNACLE (Practice INNovation And Clinical Excellence), ORBIT-AF (Outcomes Registry for Better Treatment of Atrial Fibrillation), the Paul Coverdell National Acute Stroke Registry, AFNET (the German Competence Network on Atrial Fibrillation) and regional initiatives, such as PREFER in AF (Prevention of thromboembolic events – European Registry in Atrial Fibrillation) supplement more broad-scoped registries with a localised focus, including RealiseAF (Real-life global survey evaluating patients with atrial fibrillation), RecordAF (REgistry on Cardiac rhythm disORDers assessing the control of Atrial Fibrillation) and GLORIA-AF (Global Registry on Long-Term Oral Antithrombotic Treatment in Patients with Atrial Fibrillation).

Large registries such as those described above present a number of real benefits to the various groups in society involved in antithrombotic therapy of AF patients. Healthcare systems will be able to better analyse budgetary impacts in the continuously evolving anticoagulation landscape; clinicians are aided in customising therapy trajectories to best benefit their patients, based on the myriad factors that contribute to interindividual variability (e. g. non-compliance risk factors, comorbidity profiles, renal function); the data could also be used to promote patient understanding of the various competing treatment options and their associated risks and benefits. In the Netherlands, where such awareness is still not optimal, the impact of the GARFIELD-AF registry and others of its ilk could be beneficial.
