# Erratum to: The Global Anticoagulant Registry in the FIELD – Atrial Fibrillation (GARFIELD-AF): Exploring the changes in anticoagulant practice in patients with non-valvular atrial fibrillation in the Netherlands

**DOI:** 10.1007/s12471-016-0916-5

**Published:** 2016-11-10

**Authors:** V. ten Cate, H. ten Cate, F. W. A. Verheugt

**Affiliations:** 1 Laboratory for Clinical Thrombosis and Haemostasis, Cardiovascular Research Institute Maastricht, Maastricht, The Netherlands; 2 Department of Internal Medicine, Cardiovascular Research Institute Maastricht, Maastricht, The Netherlands; 3 P.C. Hooftstraat 188, 1071 CH Amsterdam, The Netherlands

**Erratum to: Neth Heart J (2016) DOI: 10.1007/s12471-016-0874-y**

In the version of the article originally published online, there was an error in the acknowledgement section. The authors wrote: ‘and Dr. S.H.K. The (Bethesda Diabetes Research Center)’. The line should have read: ‘and Dr. S.H.K. The (Treant Zorggroep – locatie Bethesda)’.

